# Translational biomaterials of four-dimensional bioprinting for tissue regeneration

**DOI:** 10.1088/1758-5090/acfdd0

**Published:** 2023-10-09

**Authors:** Leah Faber, Anne Yau, Yupeng Chen

**Affiliations:** 1 Department of Biomedical Engineering, University of Connecticut, Storrs, CT 06269, United States of America

**Keywords:** tissue engineering, 4D bioprinting, stimuli-responsive biomaterials, biological scaffolds, self-assembling nanomaterials

## Abstract

Bioprinting is an additive manufacturing technique that combines living cells, biomaterials, and biological molecules to develop biologically functional constructs. Three-dimensional (3D) bioprinting is commonly used as an *in vitro* modeling system and is a more accurate representation of *in vivo* conditions in comparison to two-dimensional cell culture. Although 3D bioprinting has been utilized in various tissue engineering and clinical applications, it only takes into consideration the initial state of the printed scaffold or object. Four-dimensional (4D) bioprinting has emerged in recent years to incorporate the additional dimension of time within the printed 3D scaffolds. During the 4D bioprinting process, an external stimulus is exposed to the printed construct, which ultimately changes its shape or functionality. By studying how the structures and the embedded cells respond to various stimuli, researchers can gain a deeper understanding of the functionality of native tissues. This review paper will focus on the biomaterial breakthroughs in the newly advancing field of 4D bioprinting and their applications in tissue engineering and regeneration. In addition, the use of smart biomaterials and 4D printing mechanisms for tissue engineering applications is discussed to demonstrate potential insights for novel 4D bioprinting applications. To address the current challenges with this technology, we will conclude with future perspectives involving the incorporation of biological scaffolds and self-assembling nanomaterials in bioprinted tissue constructs.

## 1. Introduction

The concept of utilizing three-dimensional (3D) biological scaffolds for tissue engineering applications first emerged over two decades ago in a 1993 paper titled *Tissue Engineering* [1]. In the paper, authors Langer and Vacanti defined tissue engineering as a field that merges the fundamentals of engineering and life sciences to develop biological substitutes that improve or restore tissue function [1]. They also described how 3D biological constructs could be utilized as a scaffold to grow tissues for reparation applications [1]. Around the same time period, rapid prototyping technology, or 3D printing, was invented by the co-founder of 3D Systems, Charles Hull. He developed the first printer that had the ability to produce 3D objects by depositing photopolymers layer-by-layer and solidifying them via ultraviolet light crosslinking [2, 3]. In recent years, the field of tissue engineering has advanced immensely, and 3D bioprinting emerged into the spotlight as a means to manufacture biological scaffolds for tissue regeneration applications. The 3D bioprinting combines living cells, bioactive molecules, and biomaterials within a 3D scaffold to generate structurally functional biological constructs [4]. Although small-scale tissues have been successfully produced through bioprinting, the ultimate goal of this advancing technology is to develop multifunctional tissues and organs that can regenerate tissue defects *in vivo*. Recent advancements in the field of 3D bioprinting involve the use of bioprinted scaffolds to model various diseases and develop patient-specific clinical treatments.

In comparison to conventional scaffold fabrication methods such as salt leaching, solvent casting, freeze drying, and electrospinning, 3D bioprinting allows for the mass manufacturing of biological constructs with precise placement of cells and biomaterials [5, 6]. Salt leaching is a fabrication technique that produces porous polymeric structures suitable for cell seeding. It is commonly combined with solvent casting, where porogens are added to a polymeric solution, casted in a mold, and evaporated [7]. The subsequent leaching of the porogens leaves behind a porous microenvironment that can promote cell growth [7]. However, this is a time-consuming process that produces scaffolds with limited mechanical properties. Additionally, users have limited control over pore size and distribution. Freeze drying is a scaffold fabrication technique that involves casting a solution in a mold followed by freezing and drying steps [7]. During the primary drying step, the ice is extracted from the solution via sublimation while the remaining water is removed using a secondary drying step called desorption [7]. Although freeze drying allows for greater control over pore distribution, it is a costly process with long drying steps. Additionally, since cells can only be seeded on the scaffolds post-fabrication, this process does not allow for the homogeneous distribution of cells and bioactive molecules within the structures. Electrospinning, which produces fiber meshes using electric fields, can be used to fabricate a wide array of structures that mimic native extracellular matrix (ECM) [4]. However, like many scaffold-based fabrication techniques, it is limited in its ability to produce cell-encapsulated structures that mimic the complex microenvironment of biological tissues. An additional challenge is the ability for cells to survive following the high voltages applied during electrospinning [4].

Compared with conventional scaffold-based approaches, 3D bioprinting can be used to precisely control the location and organization of cells in a layer-by-layer process, ultimately producing homogeneously cell-encapsulated structures that more accurately mimic the structure of native tissue. Additionally, users can control the pore size and interconnectivity of the constructs using computer-assisted design (CAD) software to promote proper cell adhesion and growth within the deposited bioink [8]. Bioinks are defined as cell-based formulations used in automated biofabrication technology [9]. In order to be considered 3D bioprinting as opposed to 3D printing, the printed biomaterial has to be compatible with cell encapsulation within the structure [5]. Hydrogel-based bioinks are mainly used for 3D bioprinting since they are derived from biocompatible polymers, which mitigates the immunogenicity of the printed scaffolds [6]. Additionally, the ECM-like structural properties of soft hydrogels in combination with their ability to homogenously distribute cells throughout the scaffold promotes cell survival over long periods of time [10–12]. Ultimately, compared to conventional scaffold-based fabrication methods, 3D bioprinting enables precise spatial patterning of cells, promoting enhanced cell signaling within the fabricated constructs.

**Figure 1.**
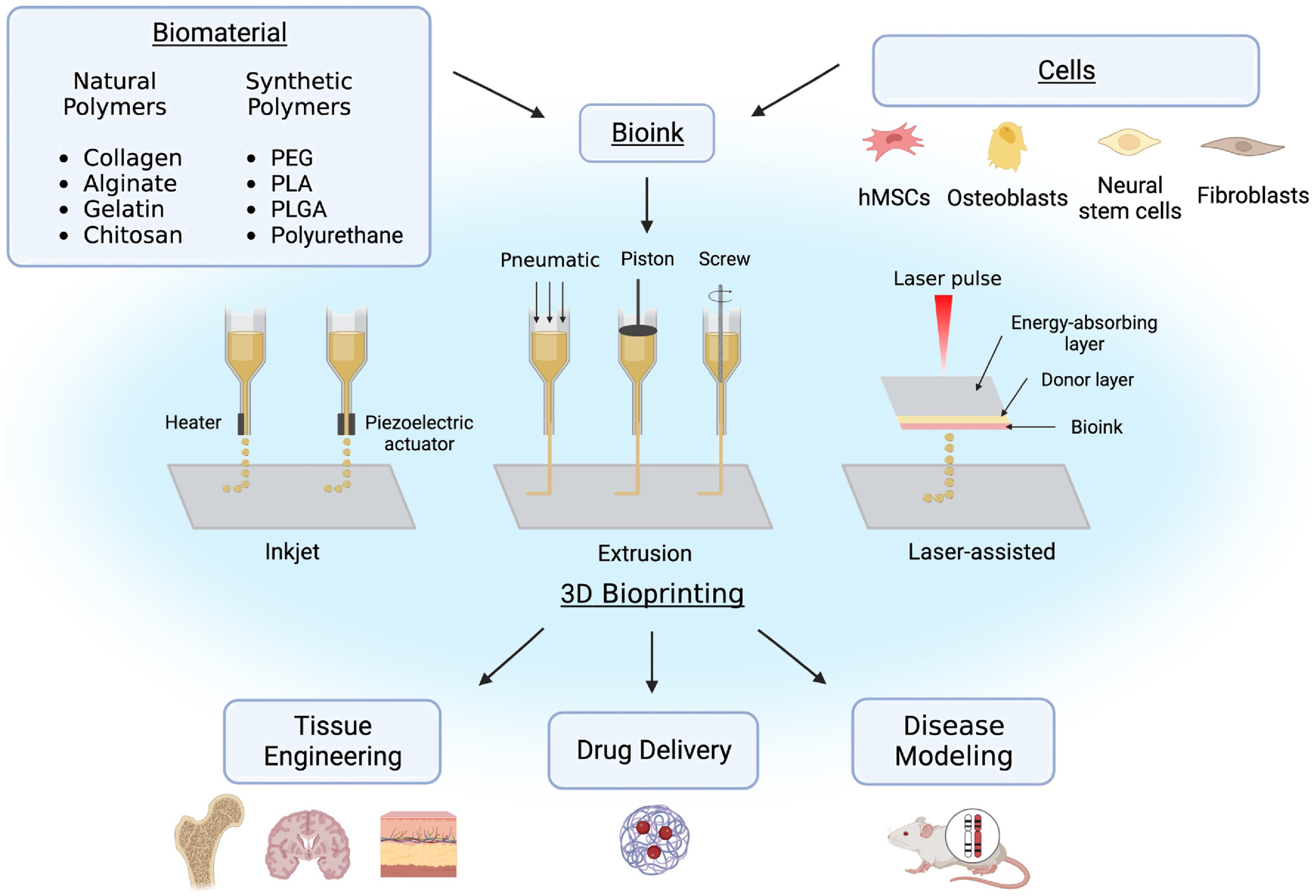
Overview of 3D bioprinting including bioink composition, common types of printing, and applications. (Image created using Biorender.com. Inspired by [13–16, 19]).

Some common types of 3D bioprinting, as displayed in figure 1, include inkjet, laser-assisted printing, and extrusion-based printing. Inkjet was the first 3D bioprinting method developed. Biological constructs produced through inkjet printing are typically created using a ‘drop on demand’ approach, in which dilute solutions are printed in individual droplets using a thermal or piezoelectric actuator [13–15]. Although it is a relatively inexpensive printing method, the increased shear and thermal stress can negatively impact the viability of cells within the constructs. Instead of a dispensing nozzle, laser-assisted bioprinting utilizes pressure generated from laser stimulation to force out droplets from a previously deposited layer of cellular or acellular biomaterial ink onto a substrate [13, 16]. Laser-assisted printing is more effective at printing more viscous materials than inkjet printing, however, it is a more complex and expensive mechanism. Extrusion-based bioprinting uses pneumatic or mechanical pressure to force a constant strandwise deposition of bioink or acellular biomaterial ink from a nozzle in a layer-by-layer fashion [13]. This type of bioprinting is the most common due to its rapid deposition rate, high shape accuracy, and reasonable cost compared to other deposition methods. Additionally, it can be used to print a wide variety of biomaterials with viscosities ranging from 10^−3^ to 10^4^ Pa*s [17]. A main challenge of extrusion-based bioprinting is the optimization of the bioink printing parameters including extrudability, strand formation, and shape stability [17]. Although a high mass flow rate is desirable for mass manufacturing, it can induce shear stress on the cells embedded within the bioink, impacting their viability [18]. Since larger cells with varied shapes are more shear-sensitive than smaller, more spherical cells, it is crucial to optimize printing parameters such as applied pressure and needle shape depending on the cells used [18]. For example, cylindrical needles typically induce larger shear stresses on the bioink compared to conical needles, where shear stress is limited to the bottom-most part of the needle [18]. Therefore, it is crucial to alter the applied pressure and needle shape depending on the cell type used to enhance cell viability within the constructs. For all three bioprinting methods, the hydrogel-based constructs are ionically or covalently crosslinked during or following its deposition to promote the stabilization of the bioprinted structures.

Printability is a common term to describe the favorability of printed materials, specifically pertaining to extrudability of the material and shape fidelity of the final product [18]. Printability of materials varies depending on the type of additive manufacturing (AM) technology used. For example, with inkjet bioprinting, printability refers to the ability of the material to form defined droplets once deposited from the dispensing nozzle. In regard to laser-assisted bioprinting, printability refers to the ability of the material to form a consistent stream once extruded, resulting in the formation of defined droplets when exposed to the substrate. Printable materials used in extrusion-based bioprinting result in consistent extruded filaments that have controllable diameter [20]. While low-viscosity biomaterials can be used for inkjet and laser-assisted bioprinting due to their droplet-based deposition approach, they are not always favorable for extrusion-based bioprinting due to its low yield stress and inability to form well-defined constructs. High-viscosity biomaterials promote enhanced shape fidelity of the deposited constructs; however, stiff materials are not a suitable environment for cell encapsulation and growth [10]. Therefore, strategies must be implemented to increase the viscosity and printability of hydrogel-based biomaterials used in extrusion-based bioprinting without enhancing the stiffness of the final constructs. For example, the viscosity of a hydrogel-based biomaterial can be increased by incorporating highly viscous biomaterials that diffuse from the scaffold into the cell culture medium post-printing. Incorporating a highly viscous biomaterial, such as methylcellulose, significantly improves the printability of the bioink while its diffusion leaves behind a low content polymer hydrogel that promotes enhanced cell growth [10].

**Figure 2.**
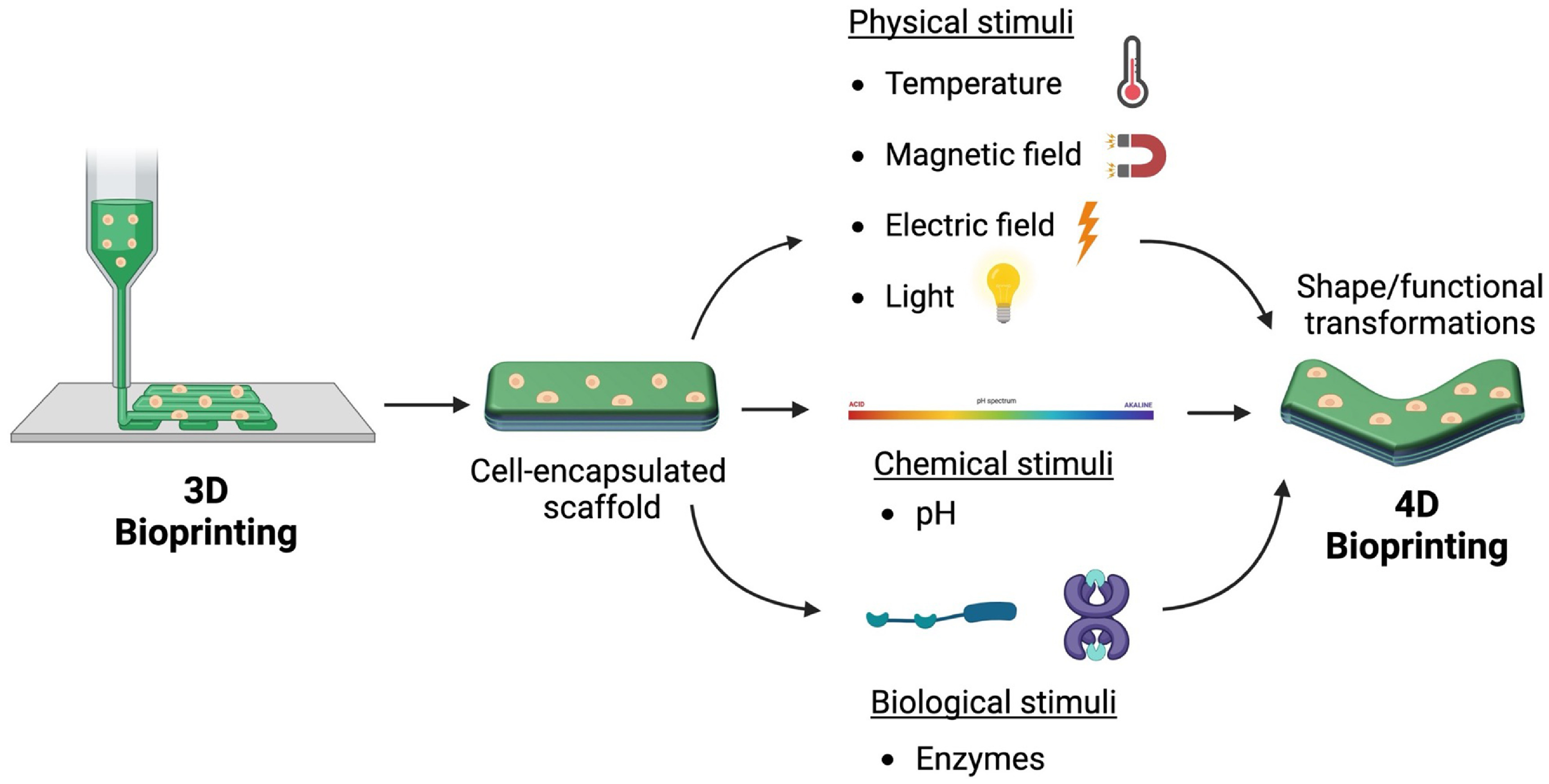
Graphical summary of 3D and 4D bioprinting of cell-encapsulated structures. (Image created using Biorender.com. Inspired by [3, 8, 12, 21].).

Although 3D bioprinting methods have been proven to successfully model the tissue architecture and stiffness of *in vivo* tissue conditions, the static nature of these constructs does not accurately model the dynamic properties of native tissue [21]. The four-dimensional (4D) printing, which combines the use of AM with stimuli-responsive materials, has emerged in recent years to develop structures that can dynamically transform their shape or function in response to physical, chemical, and biological stimuli [22]. In comparison to static fabrication technologies such as 3D printing, electrospinning, and salt leaching, stimuli-responsive materials used in 4D printing possess programmable characteristics, allowing for controlled deformations and functional changes. Additionally, dynamic 4D printed structures can possess unique features, such as the ability to self-fold, twist, and swell, ultimately resulting in complex constructs that cannot be produced using static fabrication methods [23]. Due to the versatility of 4D printing technologies, recent work has been focused on implementing these technologies within biomedical areas through the development of 4D bioprinting. The 4D bioprinting refers to the combination of cells, biological molecules, and stimuli-responsive biomaterials to develop 3D constructs that can transform structurally and/or functionally over time in response to external stimuli, as viewed by figure 2 [8]. Dynamic biological constructs fabricated using 4D bioprinting more accurately mimic the *in vivo* responses of tissues and organs compared to static constructs. Additionally, due to its controllable nature, 4D bioprinting can be used to create novel patient-specific models for tissue engineering applications and drug delivery systems. In this review, the various biomaterials and stimuli utilized in 4D bioprinting are discussed along with the wide range of tissue engineering applications. In addition, the potential for well-established 4D printing and stimuli-responsive mechanisms to be translated into 4D bioprinting processes is discussed. The review will conclude with a discussion of self-assembling nanomaterials to demonstrate potential insights for future smart nanomaterials.

## 2. 4D printing—origin and development timeline

**Figure 3.**
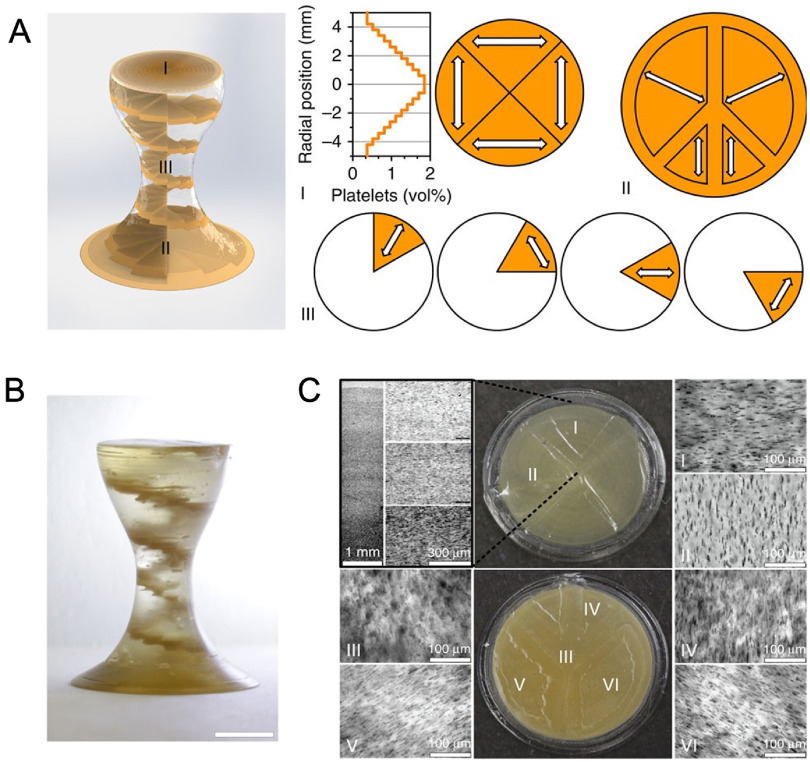
3D-printed magnetic-responsive PUA constructs doped with alumina platelets (A) the programmed design of the complexes, showing the local texture orientations (white arrows) (B) actual 3D-printed complex depicting distinct the distinct platelet helical design (C) (I, II) Top-view optical micrographs of the construct and (III–VI) bottom-view micrographs showing that the local alignment of the platelets closely matches the programmed structure. Reproduced from [26]. CC BY 4.0.

The concept of 4D printing first emerged in 2014 at the Massachusetts Institute of Technology, when Skylar Tibbits 3D printed multi-material constructs with the ability to transform over time [24]. In his demonstration, materials composing the joints of a structure were able to be expanded, subsequently changing the angle of the joints [12]. Soon thereafter, 4D printing was tested using more biocompatible materials, such as alginate and gelatin. In 2015, Bakarich *et al* 3D printed thermally responsive smart hydrogels composed of alginate and poly(N-isopropylacrylamide) (PNIPAAm) [25]. These hydrogels served as smart valves that controlled the flow of water by restricting and expanding in response to heating and cooling [25]. That same year, Kokkinis *et al* 3D printed magnetic-responsive complexes composed of polyurethane acrylate (PUA) oligomers and modified alumina platelets [26] (figure 3). The ink was deposited in precise patterns as shown in figure 3(A), with the white arrows indicating the change in local texture. A low magnetic field was applied during the printing process, ultimately aligning the brown-colored dispersed platelets in a distinct helical pattern (figure 3(B)). Optical micrograph images confirmed the success of programmed gradient, as the alignment and concentration of the platelets in each section (I–VI) closely matched those of the programmed design (figure 3(C)) [26].

Shortly after successful printing methods were developed, researchers began experimenting with cell-laden multifunctional biomaterials to mimic how native tissues respond to various stimuli. For example, in 2016, Deng *et al* developed shape memory copolymers with tunable recovery temperatures to promote the myogenic differentiation of myoblast cells seeded on top [6, 27]. Shape memory polymers (SMPs) have the ability to return to their original ‘memorized’ state when an external stimulus is applied, triggering its shape memory effects [28]. When thermoresponsive polymers are temporarily deformed, shape recovery to its permanent state is initiated at its transition temperature. The electroactive polycaprolactone-aniline trimer copolymers were created using a film casting method and C2C12 myoblasts were seeded on top of the films after their fabrication. To demonstrate the shape-memory effect of the copolymers, they were folded into a 180° shape, cooled to 4 °C to fix the temporary shape, and then immersed in water at their recovery temperature, where they reverted back to their original shape [27]. The electroactive properties of the copolymer films combined with the mechanical strain placed on the cells during shape recovery promoted myogenic differentiation of the cells, making them suitable for muscle tissue engineering models. In 2017, Hendrikson *et al* developed thermoresponsive 3D shape memory polyurethane (PU) scaffolds seeded with human mesenchymal stem cells (hMSCs) post-fabrication for stimuli-responsive tissue engineering applications [6, 28]. In this study, a thermoresponsive PU scaffold, with a transition temperature of 34 °C, was fabricated via fused deposition modeling. It was first heated to 65 °C, where a custom-made stretcher was used to apply 50% strain to the scaffolds [28]. The scaffold was then cooled to 4 °C, imparting a temporary stretched shape. Cells were seeded within the scaffold at 30 °C to allow for cell adhesion, and then the temperature was increased to 37 °C to promote shape recovery [28]. The mechanical strain placed on the cells after the material’s shape recovery directed the cells’ activity, resulting in morphological changes. This study demonstrated that SMPs can accurately mimic tissues that exhibit varying mechanical properties based on physical activity, such as muscle and bone. The ability to produce biomimetic functional constructs foreshadowed the true potential of 4D bioprinting for tissue engineering applications. Additional examples of stimuli-responsive shape memory biomaterials and their recent applications in tissue engineering are discussed in sections 3 and 4.

## 3. 4D bioprinting technology

The 4D bioprinting is an advanced form of bioprinting that combines cells with stimuli-responsive biomaterials to create complex biological structures that transform over time in response to various physical, biological, or chemical stimuli. One of the potential applications of 4D bioprinting with stimuli-responsive biomaterials is the development of implantable medical devices that can adapt to changes in the body over time with the ability to self-assemble and self-heal. For example, a 4D-bioprinted scaffold that can change its shape and size in response to changes in pressure or flow could improve outcomes for patients with tissue damages, providing structural support for cell growth and transport of nutrients [29]. Another potential application of bioprinting with stimuli-responsive biomaterials is the development of artificial organs that can respond to changes in their environment, such as changes in oxygen levels or pH, to maintain optimal function, which would be advantageous for patients requiring organ transplants. The demonstration of dynamic processes based on external triggers is the foundation of 4D bioprinting technology [30]. In this review, types of biomaterials used in 4D bioprinting are discussed in addition to the types of triggers used to create biomaterial responses.

Bioink is a cell-based formulation, typically composed of one or more biomaterials, which is used to fabricate biological constructs via bioprinting. Hydrogel-based bioinks are the most common type of bioink due to their favorable biocompatibility and ECM-like structural properties, allowing for proper cell growth and differentiation [31, 32]. The final shape takes place immediately after being bioprinted and crosslinked [33]. Currently, the most widely used natural biopolymer for bioprinting is alginate due to its low cost, non-toxic properties, and compatibility with a wide range of cell types, allowing it to be utilized for many tissue engineering applications [34]. In addition, alginate has a rapid gelation rate when exposed to divalent cations, which leads to the bridging between polymer chains and subsequent solidification [33]. The quick solidification rate of alginate is favorable for bioprinting since it decreases the total printing duration and improves the shape fidelity of the constructs [35]. However, alginate is a relatively bioinert material, limiting cell attachment. This challenge can be addressed by modifying certain adhesive peptides, such as arginylglycyl-aspartic acid (RGD) motifs, onto alginate to promote cell adhesion [33]. Another common natural biopolymer used for bioprinting is gelatin, due to its low toxicity, biodegradability, and ability to solidify at low temperatures. It is a collagen-derived protein found in native skin, tendons, and ligaments, which makes it suitable for cell attachment and growth [33]. Although gelatin forms hydrogels at lower temperatures compared to other biopolymers, temperature-induced gelation is time-consuming and can result in structural instability [33]. Therefore, gelatin is commonly modified with photopolymerizable groups and ultra-violet (UV)-crosslinked post-printing to improve the stability of the final constructs.

Synthetic biopolymers are also occasionally incorporated into bioprinting because of their favorable mechanical properties, producing constructs with high shape fidelity. However, synthetic polymers typically exhibit poor biocompatibility compared to natural polymers, limiting cell adhesion and long-term survival. More recently, cell aggregate-based bioinks, which are composed solely of cell aggregate spheroids, have been used as means to fabricate scaffold-free constructs for tissue engineering applications. Instead of self-assembling in the presence of a biological scaffold, the cells within these bioinks interact and fuse to form solid constructs [36–38]. By combining cell aggregate-based bioinks with microcarrier technology, the cells are able to adhere to the microcarriers and begin proliferating prior to printing [36]. A disadvantage of this method is that it requires a substantial number of cells, ultimately limiting the size of the final structures [36].

The biomaterial inks used in 4D bioprinting applications typically combine traditional bioprinting biomaterials with stimuli-responsive biomaterials. Shape-memory biomaterials, which are a class of stimuli-responsive biomaterials, possess programmable characteristics, ultimately allowing for the controlled deformation of the printed constructs. Common shape-memory biomaterials that are used for 4D printing are SMPs and shape memory hydrogels (SMHs) [3]. If deformed, the chemical or physical crosslinks formed within SMPs allow them to revert to their ‘memorized’ original shape when exposed to a stimulus [39, 40]. However, since cells can only be seeded on the surface of these polymers and not incorporated within a bioink formulation, they are more prone to a non-homogenous distribution, negatively affecting cell migration and proliferation [3]. SMHs are hydrogels that can change their shape and volume in response to external stimuli. The hydrogels shrink and swell in response to the reverse hydration of the polymer chains composing the hydrogel [34]. In contrast to SMPs, cells can be homogeneously distributed within SMHs prior to bioprinting, which makes them a favorable bioink for tissue engineering applications. Due to their suitable cell encapsulation properties, SMHs are preferred for 4D bioprinting applications while SMPs are mainly used for 4D printing. Both SMPs and SMHs can change their shape or functionality in response to external stimuli such as temperature, pH, magnetic attraction, and light irradiation. In the studies discussed in the following sections, many have demonstrated the ability in keeping the cells viable for their studies because the optimal conditions for cell-safe 4D bioprinting depend on the selection of appropriate materials, cell types, printing parameters, and environmental conditions. The upcoming sections will discuss common stimuli-responsive materials and how they are utilized for various 4D bioprinting applications.

### 3.1. Physical stimuli-responsive biomaterials

#### 3.1.1. Temperature-sensitive materials

Thermo-responsive materials are designed to change their physical or chemical properties at a defined temperature. As the temperature changes, the wettability and solubility of the material is altered, leading to its shape transformation [12]. One area of study is the sol–gel transformation of thermo-responsive materials once they are delivered into the human body and are exposed to the physiological temperature of 37 °C [41, 42]. Thermo-responsive materials are also being researched as potential drug-delivery vehicles, with the ability to release encapsulated drugs at predetermined temperatures [12]. Two common materials that are used for temperature-responsive applications are SMPs and responsive polymer solutions [21, 43]. Common temperature-sensitive SMPs are thermoplastic elastomers that are composed of two components: an elastic segment and a switching segment. The elastic component has a glass transition temperature while the switching component has an intermediate glass transition temperature or a melting temperature [21]. The material’s original shape is programmed at a temperature above the elastic glass transition temperature. The ability for the polymer to regain its original state is due to the elastic spring energy stored within the elastic component. If the temperature is dropped below the intermediate glass temperature and then heated again, the stored elastic spring energy is released, leading to its shape change back to its original state [21, 44]. Thermo-responsive polymer solutions are divided into lower critical solution temperature (LCST) polymers and upper critical solution temperature (UCST) polymers. LCST polymers become insoluble and undergo phase separation at temperatures above their critical temperature while UCST polymers undergo phase separation at temperatures below their critical temperature [45]. At environmental temperatures below the LCST and above the UCST, the hydrophilic and hydrophobic interactions between the polymer chains and the solvent are disrupted, which can lead to the polymer’s precipitation [45]. In terms of hydrogels, the disrupted interaction can lead to chain collapse and expansion, leading to the shrinkage or swelling of hydrogels, respectively [21]. A disadvantage of using thermo-responsive hydrogels is that temperature-induced gelation is a time-consuming process. Additionally, the rapid cooling of self-healing hydrogels can induce structure frustration, which is the result of the substantial differences in heat and solvent diffusions [46]. This ultimately results in slow diffusion of water molecules out of the hydrogel and therefore, slowed shrinking kinetics [46]. Additionally, high temperatures can negatively impact the viability of cells within bioprinted structures. A solution to this problem could be to lower the transition temperature of the thermo-responsive polymers to 37 °C through the addition of various copolymers or small nonvolatile biomolecules, eliminating exposure to high temperatures [12].

A commonly used thermo-responsive polymer solution for 4D bioprinting applications is (PNIPAAm) [47]. At external temperatures below the LCST of 32 °C, the polymer chains in PNIPAAm become hydrophilic and the material begins to dissolve [12, 48]. In a study conducted by Ozturk *et al*, a tissue engineering scaffold was produced using PNIPAAm to study how mechanical strain affects cell growth and differentiation as the construct changes shape [48]. When chemically modified with temperature-responsive elastic-like proteins (ELPs) to promote cell adhesion, it was determined that the mechanical strain experienced during temperature-invoked swelling and shrinking increased cell proliferation on the PNIPAAm scaffolds [48]. This study demonstrated the potential of using PNIPAAm films as cell carriers for tissue engineering applications. Additional examples of thermo-responsive polymer solutions include poly(N-vinylcaprolactam) and poly(ethylene glycol)-based block polymers [21].

**Figure 4.**
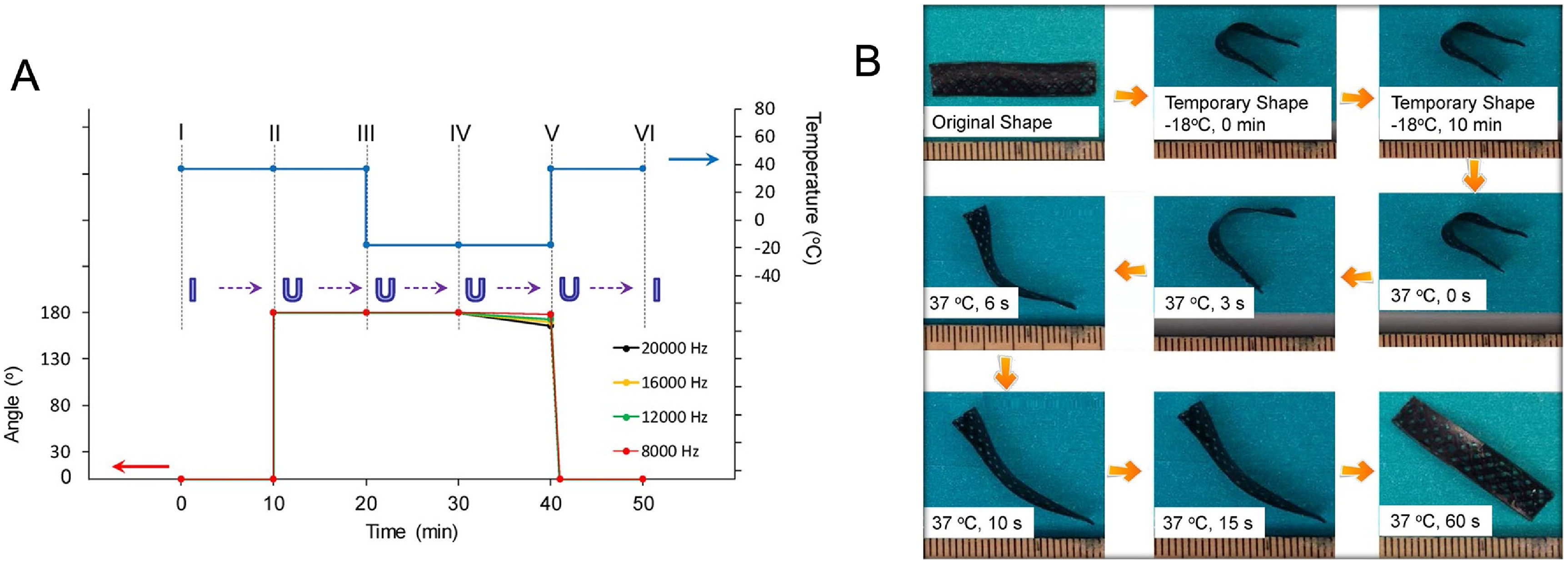
Temperature-responsive soybean oil epoxidized acrylate scaffold fabricated using 4D bioprinting. (A) Shape memory cycle at various frequencies (B) shape memory cycle demonstrated with an acellular sample. Reproduced from [50]. CC BY 4.0.

Some common examples of thermo-responsive SMPs utilized for 4D bioprinting applications are PU, soybean oil epoxidized acrylate (SOEA), and polycaprolactone triol (Ptriol) [3, 21, 49]. In a 2017 study, Miao *et al* 4D bioprinted a SOEA scaffold seeded with hMSCs [50]. The samples were first bent into a U shape at the physiological temperature of 37 °C. After assuming a straightened temporary shape when the environmental temperature dropped to −18 °C, the construct was able to revert to its original shape after the temperature was raised once again to the physiological temperature, as shown in figure 4 [50]. The biocompatible scaffolds also promoted hMSC adhesion and proliferation, which suggests its potential use for cartilage and bone regeneration applications. Shape memory PU is utilized for 4D bioprinting applications because of its wide shape memory temperature range and its great biocompatibility [49]. For example, Hendrikson *et al* produced a thermo-sensitive 3D shape memory PU structure and determined that the cells seeded within the material experienced morphology changes when exposed to the mechanical strains that accompany shape recovery [28]. These shape-memory scaffolds could ultimately be used as bioreactors for tissue regeneration. The ability to direct the behavior of cells using thermo-responsive materials holds great promise for future tissue engineering applications.

#### 3.1.2. Magnetic field-sensitive materials

Magneto-responsive materials are typically functionalized with magnetic nanoparticles (MNPs), which initiate a response in the presence of a magnetic field. The responsiveness of the MNPs results in physical and chemical changes of the polymer scaffold [21]. By varying the strength of the magnetic field, researchers can control the properties of the biomaterial, ultimately guiding the regeneration process of various tissue structures *in vitro* [51]. Magneto-responsive materials can also be utilized for site-specific drug delivery applications due to their highly manipulative nature, allowing for sustained and localized drug release. It is difficult to accurately determine the degradation rate of hydrogels; however, they can be incorporated with magneto-responsive biomaterials in order to control the release of drugs using magnetic signaling [51]. Researchers have conducted studies that analyze the drug delivery potential of magneto-responsive materials when treating heart disorders, diabetes, and various types of cancer [51, 52]. Despite their use in tissue engineering and drug delivery applications, the small size of MNPs allows them to diffuse across biological membranes, which can lead to inflammation, impaired deoxyribonucleic acid (DNA) function, and cell apoptosis [51].

One example of a magneto-responsive system utilized for tissue engineering applications is a HA/polylactide acid (PLA) scaffold incorporated with superparamagnetic nanoparticles [53]. Meng *et al* determined that when an external magnetic field was applied to the system, osteocalcin positive cell proliferation increased, inducing bone formation [53]. Fuhrer *et al* also developed a magnetic field-responsive hydrogel scaffold by suspending nanomagnets in a solution containing 2-hydroxy-ethyl-methacrylate (HEMA), ethylene glycol dimethacrylate (EGDMA), and styrene maleic anhydride copolymer (SMA) [54]. The scaffold became deformed when exposed to an external magnetic field, which induced the alignment and chondrogenesis of the seeded hMSCs by exerting a mechanical strain on them [54]. The ability for magneto-responsive materials to direct cell growth is a promising first step to ultimately regenerating entire tissues *in vitro*. To increase the biocompatibility of magnetic field-responsive systems for tissue engineering applications, MNPs are oftentimes combined with polysaccharide polymers to form magnetically responsive hydrogels that promote better cell adhesion and proliferation in comparison to synthetic polymer-based hydrogels [12].

#### 3.1.3. Electric field-sensitive materials

Similar to magneto-responsive materials, electric-field responsive materials change their shape or functionality in the presence of an electrical field. As opposed to systems limited by diffusion processes, such as thermo-responsive systems, those that respond to direct exposure to a field, such as magnetic or electric, have significantly shorter response times [55]. Hydrogels can become electro-responsive after combining with conductive polymers, such as polyaniline, polythiophene, and polypyrrole [56, 57]. When an electrical field is externally applied, ions are transported between the conductive material and the electrolyte solution, which triggers the material’s shape or size changes [12]. Although direct exposure to electric fields can negatively impact cell viability, it is suggested that a high content of conductive polymers can lower the electrical resistance across the system, mitigating the risk of overheating and subsequent cell damage [12]. Electro-responsive biomaterials that have gained recent attention in controlling stem cell growth and differentiation are carbon-based nano-biomaterials, such as graphene and carbon nanotubes (CNTs) [3, 12]. These materials have excellent electrical conductivity, controllable surface chemistry, and suitable biocompatibility which makes them favorable for nerve tissue engineering applications [58]. Their high surface area and compatibility with various functional groups allows them to be combined with different biomaterials, ultimately creating bioinks with varying chemical and physical properties [56]. In addition, the high drug loading and sustained release capabilities of CNTs makes them great candidates for targeted drug delivery applications [59].

In 2010, Lu *et al* coated CNTs with Veriflex, a shape-memory polymer, to analyze the effect that CNTs have on the polymer’s shape change and recovery [60]. The study determined that the conductivity and the speed of the polymer’s electrical response was dramatically enhanced by the incorporation of CNTs [61]. Although the research study did not analyze how cells respond to shape memory changes, it paved the way for using CNTs in biological applications. For example, in 2019, Huang *et al* bioprinted 3D porous scaffolds that were coated with multi-walled CNTs [62]. It was determined that the multi-walled CNTs enhanced human adipose-derived mesenchymal stem cell proliferation and osteoblast differentiation [62]. CNTs’ conductive and biocompatible nature has made them favorable stimuli-responsive bioink additives that allow experimenters to accurately mimic *in vivo* electrical-responsive tissues and organs.

#### 3.1.4. Light-sensitive materials

The 4D bioprinted light-responsive materials function by converting optical signals into physical and chemical responses. When exposed to an optical stimulus at high spatial resolution, the polymer chains within the material undergo photodegradation, which leads to shape deformations [3]. Like the previously discussed stimuli-responsive materials, photo-responsive materials can be utilized for both drug delivery and tissue engineering applications due to their highly tunable nature. However, the cytotoxicity of some photo-initiators can prohibit the use of select photo-responsive materials in biological applications. Near-infrared radiation is preferred over UV light for biological applications due to its ability to penetrate further into tissues [61]. In addition, its low energy level promotes enhanced cell viability within cell-laden structures [61]. Like electro- and magneto-responsive materials, electromagnetic (light) fields also promote fast response times in light-sensitive materials [63].

Although CNTs and graphene have electro-responsive properties, they are also responsive to optical signals due to their aromatic interface, which induces reversible thermal deformation [12]. Li *et al* developed single-walled CNTs combined with liquid crystal elastomer to analyze the nanocomposite’s response to optical stimuli [64]. It was determined that when exposed to white light, the films contracted up to one third of its original size [64]. However, when the light source was turned off, the nanocomposites reverted back to their original length. In addition to these materials’ ability to change shape, studies have been conducted that analyze the self-healing properties of light-responsive biomaterials. In 2018, Kuang *et al* developed a shape-memory and self-healing bioink for 4D bioprinting applications [65]. UV-light was used to cure the urethane diacrylate and linear semicrystalline polymer construct, which improved its flexibility and reparative properties. These favorable properties allowed these 3D constructs to be used as vascular repair tubes [65].

### 3.2. Chemical stimuli-responsive biomaterials

#### 3.2.1. pH-sensitive materials

pH fluctuations are indicative of normal bodily processes; however, they can also be markers of disease or infection. pH-responsive hydrogels swell and shrink in response to pH changes due to the ionization of side groups within the hydrogel [41]. The formation of ions changes the osmotic pressure, which subsequently affects the dynamic swelling behavior of the hydrogel [41]. pH-responsive polymers contain acidic or basic groups that accept or release protons in response to pH fluctuations, improving their ability to self-assemble [66]. The functional groups become charged or neutralized as the external pH fluctuates above and below a critical value, which causes the material to stretch and collapse, respectively [67]. However, this process is relatively slow compared to the response time of field-responsive materials since pH changes typically occur gradually over time [67]. Extreme pH values can also affect cell viability, therefore, stimuli-responsive materials that respond to values around physiological pH are favored to promote cell survival.

Some examples of natural pH-responsive biomaterials are chitosan, gelatin, and hyaluronic acid, while synthetic pH-responsive biomaterials include poly(l-glutamic acid), poly(acrylic acid), and poly(methacrylic acid) [21, 41]. Both synthetic and natural biomaterials have been utilized in targeted drug and protein delivery applications. For example, pH-responsive hydrogels have been studied as a means to release intravascular drugs when exposed to increased blood pH, which is indicative of cardiovascular defects [41, 68]. In 2015, Mohy Eldin *et al* developed L-arginate-grafted alginate hydrogel beads that swell and deliver proteins at a predetermined pH value [69]. Although pH-responsive materials are commonly studied as drug delivery vehicles, their ability to respond to pH fluctuations that mimic *in vivo* conditions makes them promising candidates for tissue engineering applications as well.

### 3.3. Biological stimuli-responsive biomaterials

#### 3.3.1. Enzyme-sensitive materials

Enzymes are substances that catalyze biochemical reactions. Enzyme-responsive biomaterials are designed to respond to an overexpression of specific enzymes, triggering shape memory changes [70]. Enzyme substrates induce shape changes within enzyme-responsive hydrogels by crosslinking the hydrogel or serving as a functional side group [71]. A main advantage of enzyme-responsive biomaterials used for drug delivery applications is their selectivity, preventing damage to healthy cells [70]. However, since an overexpression of enzymes is necessary in order to elicit a response, enzyme-response mechanisms are typically slow. An enzyme that has been determined to trigger the swelling and degradation of specific hydrogels is matrix metalloproteinase (MMP). Kim *et al* synthesized MMP-sensitive hyaluronic acid-based hydrogels to mimic the remodeling properties of native extracellular matrices [72]. In addition to its tunable degradation rate, the MMP-sensitive hyaluronic acid-based hydrogels also showed favorable MSC attachment and proliferation [72]. Sortase A, a bacterial ligase, was also determined to be a successful crosslinker for hydrogel-based bioinks [73]. Its quick gelation and low immunogenicity are favorable qualities for 4D bioprinting applications [73]. The rapid crosslinking, controllable degradation, and biocompatibility of enzyme-responsive hydrogels are favorable qualities for tissue regeneration scaffolds.

### 3.4. Multi-responsive biomaterials

Multi-responsive biomaterials are sensitive to a combination of stimuli. Commonly used combinations of stimuli-responsive materials include temperature and pH, magnetic field and temperature, and pH and magnetic field. Multi-responsive biomaterials have the potential to revolutionize the field of tissue engineering by enabling the creation of structures that can mimic the properties of natural tissues by responding to multiple types of environmental stimuli. For example, Li *et al* fabricated PAA-based thermosensitive and pH-sensitive hydrogels for cardiac tissue models via copolymerization [74]. These hydrogels solidified at the pH of an infarcted heart (pH of 6–7) at physiological temperature but could not solidify at the pH of blood (7.4) at the same temperature [74]. Similarly, Kanaan *et al* synthesized chitosan/ionic liquid-based hydrogel networks that were responsive to electric fields, ionic strength, and pH for advanced wound dressing applications [75].

While multi-responsive biomaterials offer many advantages for 4D bioprinting, there are some potential disadvantages that should be considered. For example, the fabrication process of these materials can be complex and they may have a limited range of responses [76]. The type of multi-responsive biomaterials may need to be carefully selected for *in vitro* studies due to the variability of the stimuli, in which complexity, biocompatibility, and response range can negatively impact cell viability, limiting their use in 4D bioprinting applications [12]. Despite these potential drawbacks, researchers continue to explore the use of multi-responsive biomaterials for 4D bioprinting. Further research is needed to better understand the limitations of these materials and to develop strategies to overcome these challenges. With continued development and innovation, multi-responsive biomaterials have the potential to revolutionize 4D bioprinting and enable the creation of complex, functional tissues for a wide range of biomedical applications.

## 4. Applications of 4D bioprinting in tissue engineering

The 4D bioprinting has advanced in recent years as a novel model for tissue engineering applications. The following section will discuss previously developed 4D bioprinting models that have been utilized for a variety of tissue engineering applications including musculoskeletal, cardiovascular, nervous, and skin tissue repair. However, due to the novelty of 4D bioprinting and the limited number of current 4D bioprinted models, both 4D printing mechanisms and smart biomaterials that are used for tissue engineering applications are also mentioned. These mechanisms serve as promising insights for future 4D bioprinting applications and are considered relevant to the scope of the review.

### 4.1. Musculoskeletal tissue applications

The musculoskeletal system protects bodily organs, controls movement, and maintains bodily support and stability. It is composed of bone, skeletal muscle, cartilage, ligaments, and tendons [77]. Although many of these tissues can self-heal minor injuries, severe damage may require surgical intervention [78, 79]. The long-term goal of 4D bioprinting for musculoskeletal tissue engineering applications is to fabricate stimuli-responsive biological scaffolds that can be used to enhance tissue regeneration at the injury site, reducing the need for autografts or allografts. When replicating these types of tissues via 4D bioprinting, it is important to accurately mimic their native dynamics using proper stimuli-responsive biomaterials.

**Figure 5.**
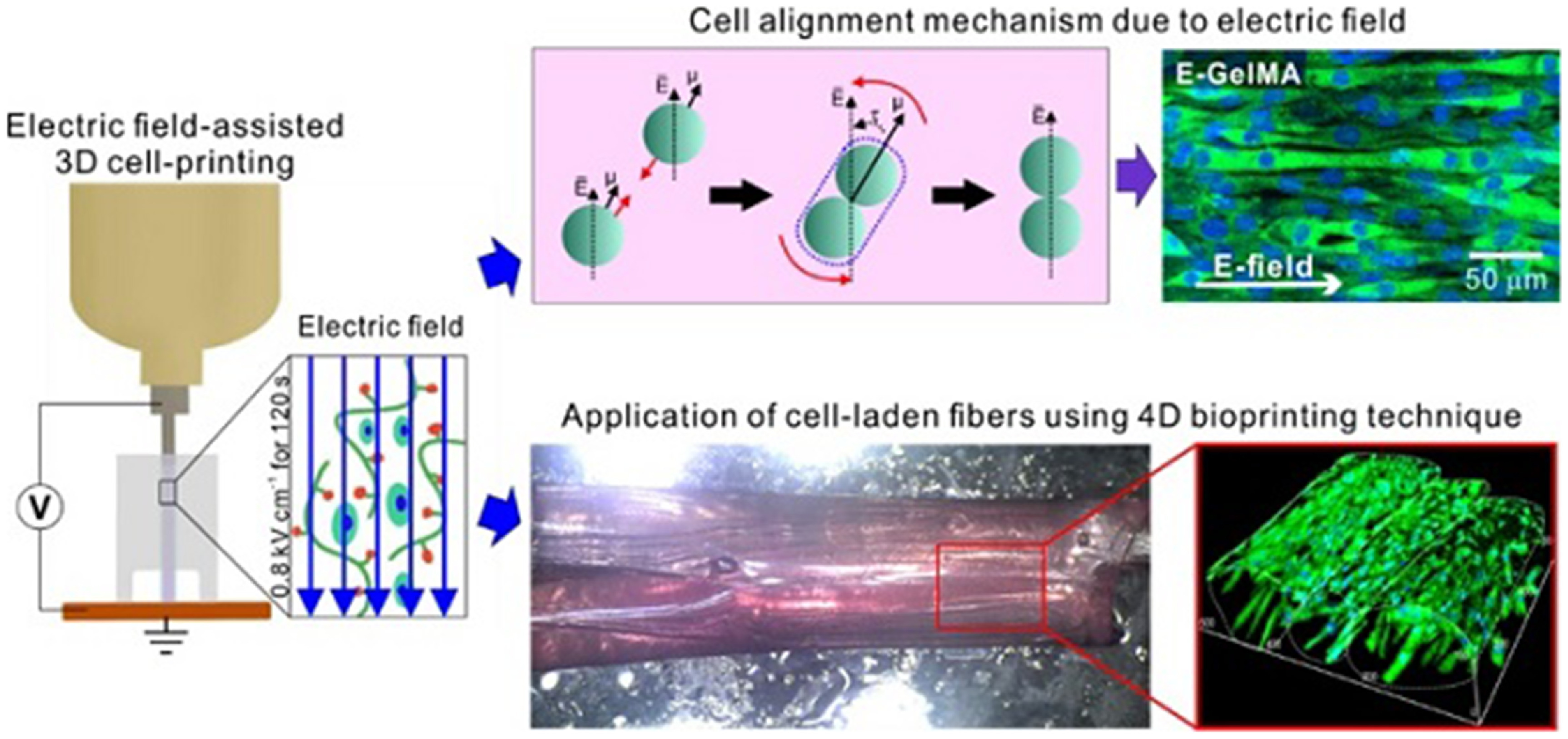
Illustrative diagram of a skeletal muscle model using GelMA-based cell-aligned bioink processed with electric-field assisted bioprinting. Reproduced from [80]. CC BY 4.0.

Skeletal muscles are very dynamic tissues. They not only act to produce controlled, voluntary body movements, but they also are able to self-heal minor tears or strains [80, 81]. Recent advances in tissue engineering include the use of 4D bioprinting to fabricate synthetic skeletal muscle tissue that can repair more serious musculoskeletal defects *in vivo*. In early 2021, Yang *et al* bioprinted a 3D gelatin methacryloyl (GelMA) scaffold encapsulated with C2C12 cells, a subclone of myoblasts derived from mouse skeletal muscle, to study its effectiveness in inducing myoblast differentiation [80, 82]. An electrical field was applied during the bioprinting process, which allowed for proper alignment and differentiation of the C2C12 cells within the GelMA scaffold, as visualized in figure 5 [80]. The alignment of these cells led to myotube formation and maturation. Cell alignment is crucial when developing accurate 3D bioprinted muscular tissue models in order to mimic the highly organized structure of native muscle fibers. After electrical stimulation, the structure was crosslinked with UV light, which allowed for greater stability and the development of a 4D gelatin film. The gelatin film was then placed in a culture medium, where it swelled and began to roll up the GelMA fibers into bundles that simulated fascicles [80]. The ability for these cell-laden GelMA scaffolds to form fascicle-like structures is a promising step in the field of musculoskeletal tissue engineering.

Stimuli-responsive biomaterials that promote the osteogenic differentiation of stem cells have been studied as candidates for personalized bone repair applications. Prior to the emergence of 4D bioprinting, stimuli-responsive biomaterials that promoted bone tissue regeneration *in vitro* were still studied. For example, in 2013, Dessi *et al* produced a temperature-responsive chitosan-based hydrogel crosslinked with *β*-glycerophosphate [83]. The hydrogel transitioned from a liquid to gel state at the physiological temperature of 37 °C [83]. The nanocrystal structure of the construct also accurately mimicked the chemical and physical properties of native bone tissue, allowing for proper adhesion and proliferation of MG63 osteoblast-like cells [83]. The analysis of stimuli-responsive biocompatible materials such as these helped pave the way for future 4D printing and bioprinting studies. In 2014, Zhang *et al* designed a 4D-printed mesoporous bioactive glass/polycaprolactone scaffold embedded with magnetic Fe_3_O_4_ nanoparticles [84, 85]. Not only did the scaffolds present similar compressive strength to native bone, but the magnetic-responsive nanoparticles induced heating of the structure when exposed to magnetic stimulation. The magnetic heating ability of the smart structures stimulated proliferation, osteogenic gene expression and ECM mineralization of human bone marrow-derived mesenchymal stem cells embedded within the constructs [85]. This magnetic-responsive scaffold was utilized for bone regeneration applications. Due to the mechanical strength of bone tissue, there are a limited amount of bone tissue engineering 4D bioprinting studies that use soft hydrogels to encapsulate cells within the structures. However, there have been studies that have designed stimuli-responsive hydrogels for bone tissue engineering applications using other conventional scaffold fabrication methods. For example, in 2018, Xu *et al* fabricated a pH-responsive chitosan/tripolyphosphate hydrogel using injection molding [86]. The pH-responsive resorption due to the regulated primary amine content allowed for the structure to absorb or release water when exposed to pH changes [86]. The swelling also stimulated proliferation of the seeded bone marrow mesenchymal stem cells, ultimately making this scaffold suitable for bone regeneration applications. Further work in this field lies in converting similar stimuli-responsive biomaterials into bioink to be used for 4D bioprinting studies.

**Figure 6.**
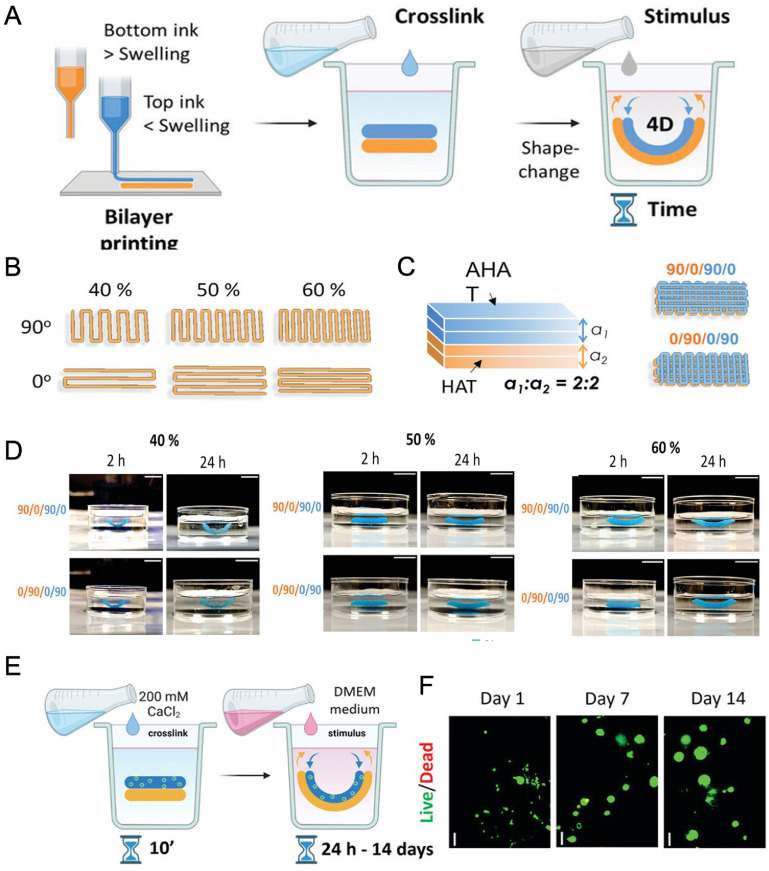
Swelling-dependent shape transformation of 4D bioprinted constructs for cartilage tissue engineering (A) schematic of the fabrication process. High swelling and low swelling inks are printed a bilayer, crosslinked, and a swelling-based stimulus is applied on the construct. (B) The printing pattern designs with different printing angles (90° or 0°) and different infill densities (40%, 50%, or 60%). (C) Schematic showing the four sublayers; two top layers of AHAT and two bottom layers of HAT. (D) Images of the scaffolds printed at various infill densities and printing angles at 2 and 24 h after the stimulus was applied. (E) Schematic of the shape-transformation process of 4D bioprinted scaffolds containing hMSCs. (F) Live/dead fluorescent images of the cell-laden scaffolds after 1, 7 and 14 d of stimulus application. Reproduced from [93]. CC BY 4.0.

Cartilage tissue engineering has advanced in recent years in an attempt to address the shortcomings of current cartilage defect treatments [87]. There are a couple surgical options that can be used to repair hyaline articular cartilage defects; however, many are short-lived due to the production of fibrocartilage tissue, which is mechanically inferior to hyaline cartilage [88, 89]. Additionally, the avascular nature of cartilage makes it nearly impossible for it to regenerate naturally [90, 91]. Therefore, tissue engineering has been studied as a long-term treatment plan for cartilage defects. Betsch *et al* developed a magneto-responsive 4D-bioprinted multilayered biological construct that accurately mimicked the dynamics of native cartilage [92]. The bioink was composed of low gelling temperature agarose, type I collagen, and streptavidin-coated iron nanoparticles [92]. A magnetic field was applied to the printed constructs, which aligned the collagen fibers, ultimately increasing their compression moduli [92]. In addition, the aligned fibers aided in stem cell adhesion and proliferation, showing great promise for cartilage tissue engineering applications. In 2022, Díaz-Payno *et al* 4D bioprinted swelling-dependent hMSC-laden constructs for cartilage tissue engineering applications (figure 6) [93]. High-swelling tyramine-functionalized hyaluronan (HAT) ink composed the bottom layer of the bilayer structures while low-swelling alginate with HAT (AHAT) composed the top layer. After crosslinking with CaCl_2_, the constructs were exposed to an aqueous solvent stimulus (solution containing 0.9% NaCl and 2 mM CaCl_2_) [93]. The differential swelling between the two layers promoted the scaffold’s self-bending. Once the printing parameters were optimized, including printing angles and infill density, hMSCs were incorporated into the AHAT bioink and Dulbecco’s Modified Eagle Medium (DMEM)-based chondrogenic media was used as the swelling-based stimulus. High cell viability, cartilaginous matrix deposition, and sustained curvature was observed after 28 d of culture [93]. These complex, multilayer 4D scaffolds could ultimately be used to mimic the curvature and cell density of native cartilage.

### 4.2. Cardiovascular tissue applications

Cardiac tissue has poor self-healing properties due to the inability of cardiac myocytes to divide and replace damaged cells [94]. Instead of regenerating the damaged tissue, scar tissue forms, which limits the contractile properties of the tissue and can lead to heart failure [94]. Tissue engineering has emerged as a novel way to regenerate cardiac tissue without the formation of scar tissue, preventing cell necrosis or apoptosis. As previously discussed, Miao *et al* successfully developed a thermo-responsive SOEA scaffold for cartilage and bone tissue engineering applications [50]. Miao and his coworkers continued to study the regenerative properties of this type of scaffold, and in 2018, developed a 4D-printed smart SOEA scaffold using photolithographic-stereolithographic-tandem fabrication (PSTS) [95]. PSTS is a technique that can produce 4D hierarchical micropatterns, ultimately promoting cell growth and alignment [95]. The construct also demonstrated shape memory properties and distinctive cardiomyogenesis of hMSCs when exposed to an external stimulus (immersion in ethanol), indicating its potential for future cardiac tissue regeneration applications. In 2017, Xu *et al* injected a temperature-responsive chitosan hydrogel into rats with myocardial infarction to promote cardiac tissue regeneration [96]. The scaffold increased MSC retention and successfully promoted cardiomyocyte (CM) differentiation [96]. The differentiation of the MSCs aided in blood vessel formation, ultimately enhancing cardiac function in the diseased rat models. Although the scaffold was not bioprinted, chitosan is a common naturally-derived bioink, therefore this study could be easily translated into 4D bioprinting applications.

Since heart contractions are regulated by an electrical conduction system *in vivo*, researchers have also studied the use of electro-responsive 3D-printed biomaterials for cardiac tissue engineering applications. In 2017, Dong *et al* fabricated electroactive hydrogel scaffolds through microextrusion 3D printing [97]. These scaffolds were composed of Pluronic F127, a copolymer surfactant, and aniline tetramer-grafted polyethylenimine (AT-PEI) [97]. The AT, when conjugated, significantly improved the material’s electroactivity [97]. Although cells were not seeded within the scaffolds, Pluronic F127 is a biocompatible hydrogel that has been widely used for drug delivery and cell encapsulation applications, making it a good candidate for bioprinting studies. In addition, the electrical activity of the F/AT-PEI constructs makes it a suitable scaffold for 4D bioprinting applications. For example, when exposed to an electric field, the highly conductive F/AT-PEI scaffolds could transduce electrical energy into mechanical work, resulting in size or shape changes that would stimulate cell proliferation and differentiation. The ultimate goal of this study is to use the fabricated scaffolds for cardiac nerve regeneration applications since cardiac tissue is sensitive to electrical stimulation [97].

**Figure 7.**
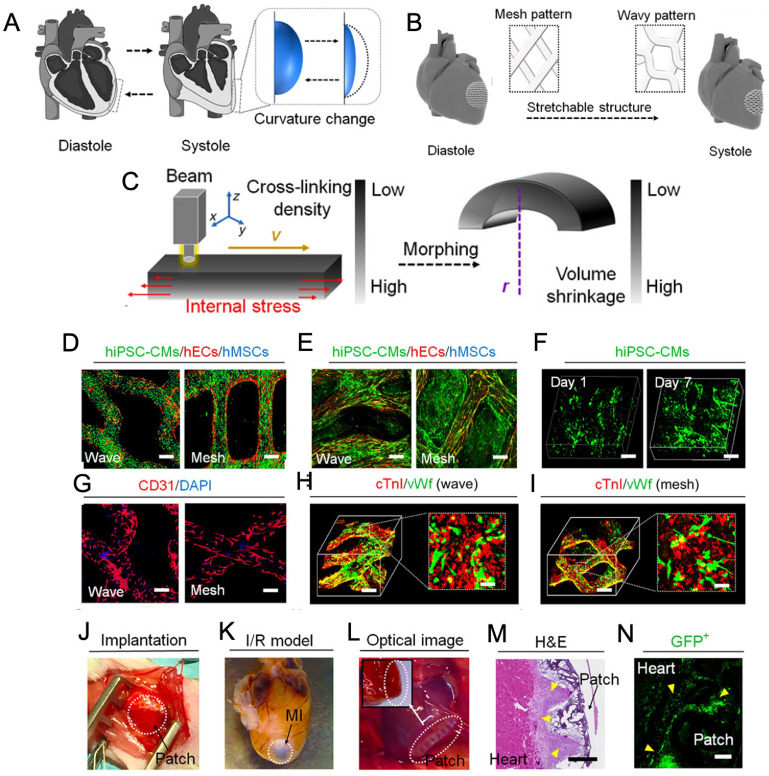
4D bioprinted scaffolds used for cardiovascular tissue engineering model. (A) Curvature of the heart at two different phases of the cardiac cycle. (B) CAD design of internal architecture (mesh or wavy) that could adapt to the movements of the cardiac cycle. (C) The internal stress-induced morphing process of the constructs as a result of variable crosslinking density using beam-scanning SLA. Cell distribution of hiPSC-CMs (green), hECs (red), and hMSCs (blue) on the cardiac patch after (D) 1 d of confluence and (E) 7 d of confluence. (Scale: 200 *μ*m) (F) Autofluorescence 3D images of hiPSC-CMs on the wave-patterned patch. Immunostaining of (G) capillary-like hEC distribution (red) on the patches and (H)–(I) cardiac-troponin-I (cTnI) (red) and von Willebrand factor (vWf) on the wave and mesh-pattern patches. (Scale: 200 *μ*m) (J)–(K) *In vivo* implantation of 4D cardiac patches in a murine model with ischemia-reperfusion injury. (L) Optical image of implanted patch at week 3 with respective (M) H&E image and (N) Fluorescent image of hiPSC-CMs showing high engraftment rate as indicated by the yellow arrows. (Scale: 100 *μ*m). Reproduced with permission from [98]. CC BY-NC 4.0.

Replicating the surface curvature of the heart is also crucial in order to accurately mimic the function of the heart in cardiac tissue models. For example, Cui fabricated a light-sensitive 4D cardiac patch using a beam-scanning stereolithography (SLA) bioprinting technique for the treatment of myocardial infarction (figure 7) [98]. The ink was composed of GelMA, a photocurable biomaterial, and polyethylene glycol diacrylate (PEGDA), which was used to increase the structural stability of the printed hydrogels. The light-induced graded internal stress in combination with a solvent-induced relaxation of the material resulted in 4D conformations of the fabricated constructs that mimicked the surface curvature of the heart [98]. The autonomous shape morphing also directed the fiber pattern from a flat mesh pattern to a 4D wavy pattern, replicating the structure of cardiac muscle fibers during diastole and systole. By triculturing CMs (hiPSC-CMs), hMSCs, and human endothelial cells (hECs), the constructs were able to successfully replicate both the anisotropic fiber structure and vascular network of native heart tissue. Compared to cell-laden samples, the cells seeded on the scaffolds post-printing showed higher proliferation and contraction rate [98]. When implanted in a murine model with ischemia-reperfusion injury, histological examination showed a decrease in infarct size compared to the control group, demonstrating its potential for use in cardiac tissue engineering applications.

### 4.3. Nervous tissue applications

The brain, spinal cord, and nerves are all composed of nervous tissue. They allow the body to communicate by sending and receiving electrical and chemical signals. The 4D bioprinting has emerged to develop smart biomaterials that can fold when exposed to various stimuli, simulating the cortical folding *in vivo* that gives the brain its wrinkled topology [99]. In addition, studying the folding process via 4D bioprinting can help researchers understand how mechanical stresses impact neural cell development. Hydrogels are typically utilized for neural tissue 4D bioprinting applications since they have favorable folding capabilities and promote the growth and differentiation of neural stem cells (NSCs) [99]. There are many limitations that accompany present-day nervous system disorder treatments, as many are meant to solely relieve pain and not cure the disease [100]. The 4D bioprinting and its ability to produce stimuli-responsive nervous tissue is slowly emerging as a novel field that could revolutionize the treatment of such disorders.

Miao *et al* utilized SLA-based 4D printing to develop graphene-based reprogrammable constructs for neural engineering applications [101]. Graphene is a favorable material for nerve tissue engineering due to its high electron mobility, excellent conductivity, and ability to promote neural cell differentiation [101, 102]. The thermomechanical shape memory characteristics of these tubular constructs allowed for them to be temporarily opened, which is a favorable characteristic for potential *in vivo* surgical implantations [101]. More specifically, the constructs, which were fixed in a flattened shape, resumed their tubular structure when exposed to physiological temperature, successfully serving as a nerve guidance conduit. In addition, varying the amount of graphene nanoparticles within the structures resulted in fluctuating light-induced graded internal stress, impacting the degree of curvature of the construct [101]. Adding more graphene nanoparticles prevented the laser from completely penetrating the construct during the printing process, resulting in weak crosslinking. Weak crosslinking ultimately decreased the thickness of the structures, subsequently increasing the degree of curvature. To assess the biocompatible nature of the constructs, hMSCs were seeded onto them and exposed to neural differentiation medium. The dynamic 4D constructs promoted cell alignment and neural cell differentiation, successfully replicating the highly organized nature of neural tissues [101]. Once 4D printing studies such as these were optimized, 4D bioprinting studies, in which cells were directly encapsulated within bioink, became more prevalent. For example, Hsieh *et al* bioprinted thermoresponsive NSC-encapsulated hydrogels made up of water dispersed PU (WDPU) nanoparticles that self-assembled into layered structures at 37 °C [103]. The structures not only promoted neural cell differentiation but were also able to assemble without the use of harmful chemical crosslinkers [101]. The measured elastic moduli of the constructs were very similar to that of brain tissue, making them promising candidates for brain tissue modeling and regeneration applications [103]. Future work in the field of brain tissue modeling lies in accurately mimicking the blood brain barrier, which has proven to be difficult due to its high selectivity and complex structure [104].

### 4.4. Skin and wound healing applications

Skin is highly prone to injury due to its direct exposure to the external environment [105]. Various types of skin-related wounds include punctures, lacerations, and abrasions. Burns are one of the most common types of skin-related trauma, affecting millions each year. In severe cases, grafting is required to reconstruct the skin in the affected area. Current skin substitutes lack the cellularity of native skin tissue, increasing the chances of a negative immunological response [106]. Bioprinting allows for the precise placement of cell types within the printed construct and can be used to fabricate biocompatible skin substitutes that accurately mimic the composition and functionality of native skin tissue [105].

Although multiple individuals have successfully 3D bioprinted biological constructs similar in composition to native skin tissue, there have been few that utilize stimuli-responsive materials in order to mimic skin’s dynamic, healing nature. Ng *et al* bioprinted a polyelectrolyte gelatin-chitosan hydrogel seeded with fibroblasts (FBs) that holds promise for future skin tissue engineering applications [107]. Chitosan is a biomaterial that is favorable for wound-healing applications due to its hemostatic nature and its ability to prevent bacterial growth [107]. The opposing functional groups within the chitosan and gelatin modified the chitosan to form polyelectrolyte complexes [107]. Polyelectrolytes are highly sensitive to electrical fields due to their electrostatic nature. Ultimately, the interaction of the negatively charged gelatin and positively charged chitosan created a suitable charge density that promoted cell attachment and proliferation [107]. By electrically stimulating the printed structure, the cell adhesion and functionality of the constructs could potentially further improve, more accurately mimicking the dynamic nature of native skin tissue. Due to the dynamic pH of skin wounds throughout the healing process, many studies have analyzed the use of pH-sensitive biomaterials to promote skin regeneration. For example, Zhu *et al* developed pH-sensitive methacrylated chitosan hydrogels seeded with NIH/3T3 FBs to be used as dynamic wound dressings [108]. The hydrogels were formed via chain and step-growth polymerizations. The biocompatible hydrogels were determined to have tunable mechanical properties, swelling ratios, and pH sensitivities, ultimately allowing for stage-specific skin regeneration [108]. Chitosan-based bioinks are favorable due their good printability at physiological temperature and high shape fidelity, therefore, this biomaterial could be a suitable candidate for 4D bioprinting applications.

An important aspect when replicating native skin tissue for clinical applications is vascularization, as it promotes proper nutrient and oxygen supply. The 4D bioprinting can be utilized to create dynamic, vascularized biological constructs. In 2020, Baltazar *et al* developed 3D bioprinted multilayered vascularized skin grafts composed of two bioinks [109]. The first bioink, which produced the dermis of the structure, was composed of human foreskin dermal FBs, hECs and human placental pericytes (PCs) suspended in type I collagen [109]. The second bioink, which formed an epidermis, was composed of human foreskin keratinocytes (KCs) [109]. When combined, the ECs and PCs self-assembled into vascularized networks and promoted KC proliferation. When implanted into mouse models, the vascularized bioprinted constructs successfully integrated with the mouse microvessels to form skin grafts [109]. In comparison to the nonvascularized skin grafts, skin substitutes incorporated with human ECs and PCs also showed a higher degree of epidermal organization and early formation of rete ridges, which are interdigitations of the epidermis and dermis [109]. Vascularized skin grafts have shown increased retention and survival in comparison to avascular grafts and have revolutionized the field of skin tissue engineering. By incorporating stimuli-responsive biomaterials into the bioink, this study could be translated into 4D bioprinting. For example, stimuli-responsive biomaterials could be used to initiate structural changes at physiological temperature that would promote the assembly of ECs and PCs into vascularized networks. The controllable nature of 4D bioprinting is ultimately used to create dynamic biological structures that mimic native tissue functionality.

## 5. Current challenges

**Figure 8.**
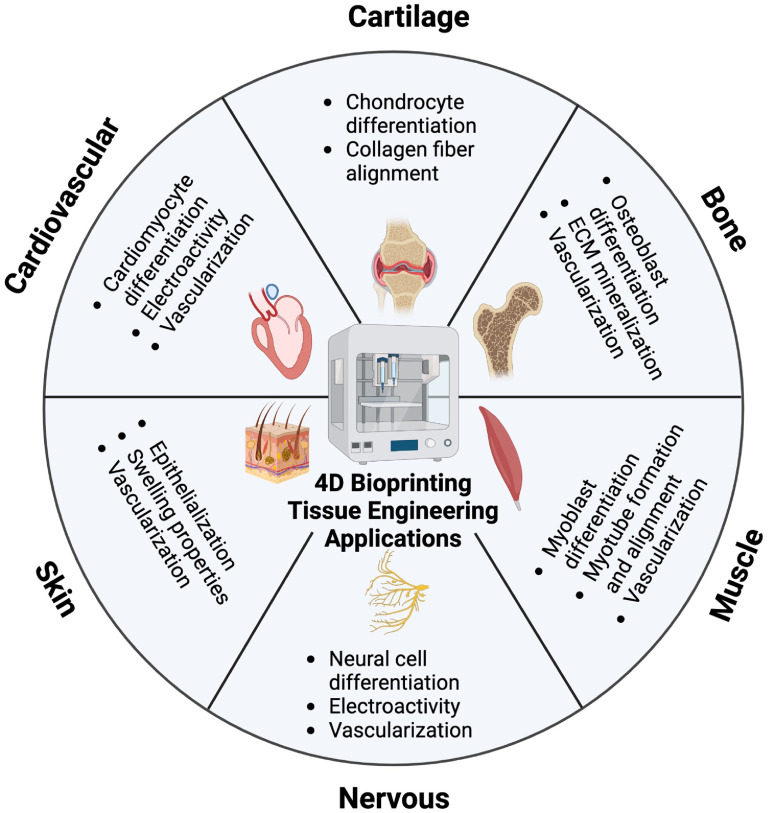
4D bioprinting tissue engineering applications and dynamic properties of each tissue type initiated via external stimuli. (Figure created using Biorender.com).

Ultimately, stimuli-responsive biomaterials can be used to fabricate dynamic tissue constructs, as illustrated by figure 8 and table 1. The 4D bioprinting, which combines 3D bioprinting with stimuli-responsive biomaterials, is rapidly emerging as the next generation of tissue engineering. Compared to conventional scaffold fabrication techniques, 3D bioprinting allows for precise placement of cells and bioactive molecules within the constructs, more accurately mimicking the cellular structure of native tissues [4, 5]. Additionally, the shape and functional changes of 4D bioprinted constructs can be used to simulate the functionality of various tissues *in vivo*.

**Table 1.** Stimuli-responsive biomaterials and their applications in tissue engineering.

Stimulus	Biomaterials used	Cell type(s)	Stimuli-responsive mechanisms	Application	References
Temperature	PNIPAAm films chemically modified with ELP	Bone marrow MSCs	Swelling properties	Bone tissue engineering	[48]
	Soybean oil epoxidized acrylate	hMSCs	Shape changes	Cartilage tissue engineering	[50]
	Polyurethane	hMSCs	Shape changes	Bone, muscle, and cardiovascular tissue engineering	[28]
	Chitosan hydrogel	hMSCs	Swelling properties	Cardiac tissue regeneration	[96]
	Water dispersed polyurethane-based hydrogel	Neural stem cells	Shape changes	Neural tissue engineering	[103]
Magnetic field	nHA/PLA scaffold incorporated with superparamagnetic *γ*-Fe_2_O_3_ nanoparticles	Osteoblasts	Superparamagnetic properties	Bone tissue formation and remodeling	[53]
	Magnetic hydrogel composed of HEMA, EGDMA, and SMA	hMSCs	Magnetic-responsive cyclic deformation	Cartilage tissue engineering	[54]
	Bioactive glass/polycaprolactone scaffold embedded with magnetic Fe_3_O_4_ nanoparticles	Human bone-marrow derived MSCs	Magnetic hyperthermia	Bone tissue engineering	[85]
	Low gelling temperature agarose and type I collagen embedded with streptavidin-coated iron nanoparticles	hKACs, hMSCs	Magnetic-responsive properties	Cartilage tissue engineering	[92]
Electric field	GelMA	C2C12	Electric-field induced cell alignment, swelling properties	Muscle tissue regeneration	[80]
	Pluronic F127, aniline tetramer-grafted polyethyleneimine	N/A	Electro-responsive properties	Candidate for cardiac and nerve tissue regeneration	[97]
Light	Urethane diacrylate and linear semicrystalline polymer	N/A	UV curing	Vascular repair tubes, soft robotics	[65]
	GelMA and PEGDA	hiPSC-CMs, hECs, hMSCs	Light-induced graded internal stress combined with solvent-induced relaxation of the material	Cardiac tissue engineering	[98]
	Graphene nanohybrid	hMSCs	Light-induced graded internal stress with subsequent shape changes	Nervous tissue engineering	[101]
pH	Chitosan hydrogel	NIH/3T3 fibroblasts	Swelling and mechanical properties	Dynamic wound dressings	[108]
Enzymes	MMP-sensitive hyaluronic acid-based hydrogel	hMSCs	Enzyme-responsive degradation	Tissue remodeling and regeneration	[72]
	Hyaluronan modified with SA-substrate peptides	Human chondroprogenitor cells, human embryonic kidney cells	Enzymatic crosslinking	3D screening platforms, tissue engineering	[73]

Although there have been great advantages in the field of bioprinting, there are still many challenges that have halted widespread use for clinical applications such as the complexity of the materials and the cell biocompatibilities with said materials. Developing bioinks that accurately mimic the mechanical and biochemical properties of native tissues remains a critical hurdle. Additionally, scaling up the technology for mass production and addressing regulatory and ethical considerations for clinical applications are essential challenges that need to be carefully navigated. Creating complex biological structures with multiple cell types and intricate structure remains a significant challenge. Current bioprinting technologies struggle to precisely recreate the complex microenvironments found in natural tissues and organs. Additionally, it is difficult to optimize the printability and biocompatibility of stimuli-responsive biomaterials [3]. Although various stimuli-responsive biomaterials have been successfully used as scaffolds for tissue engineering applications, finding a suitable bioink viscosity for printing remains a challenge. Difficulties also exist when designing a bioink that can sustain the high pressures experienced during the printing process and still maintain its stimuli-responsive nature post-fabrication.

The poor cytocompatibility of most stimuli-responsive biomaterials combined with the harsh stimuli experienced during the 4D bioprinting process can also negatively impact the viability of the cells embedded within the structures. The maintenance of cell viability within 4D bioprinted structures is essential to ensure that the constructs remain functional and viable even after being subjected to external stimulation such as light, pH or electrical stimulation. Several studies have investigated the effects of various printing parameters and biomaterials on cell viability in 4D bioprinting. Firstly, it is crucial for the cells to remain viable after exposure to the shear stresses experienced during the printing process. Cidonio *et al* (2020) found that the incorporation of nanoclay particles into bioink improved cell viability and enhanced the mechanical properties of printed structures [110]. Nanoclay-based formulations are gaining attraction for bioprinting applications due to their ability to shield encapsulated cells from shear-induced damage during the extrusion process, enhancing their viability [110]. For 4D bioprinting applications, stimuli-responsive materials that lack bioactivity can be modified with copolymers and/or bioactive peptides to improve cell adhesion and viability. Gugulothu and Chatterjee reported that the use of bioink composed of GelMA and poly(ethylene glycol) dimethacrylate improved cell viability and proliferation compared to other bioink formulations, even when combined with a photoinitiator to initiate light-responsive shape changes [111]. By varying the fraction of the two polymers, the formulation that promoted enhanced cell viability was able to be optimized. Additionally, thermo-responsive polymers with high transition temperatures can be modified by adding copolymers to reduce the transition temperature to around physiological temperature, enhancing cell viability [12]. Challenges maintaining cell viability can also negatively impact the overall functionality of dynamic structures, such as vascularization. Material modifications such as those mentioned are necessary to improve the biocompatibility of stimuli-responsive biomaterials.

Another main limitation of 4D bioprinting is that most stimuli-responsive biomaterials only perform simplistic shape changes and respond to one type of stimuli, which is not representative of the complex and diverse stimuli *in vivo* [23]. Therefore, optimizing the design of biocompatible multi-responsive biomaterials is crucial when developing 4D bioprinted *in vitro* models that accurately model *in vivo* processes. Although process and material modifications must be made before 4D bioprinting can be widely used for practical applications, it is worth noting that the field of bioprinting is rapidly evolving, and researchers are actively working to overcome these limitations. Continuous advancements in material science, bioink development, and tissue engineering techniques are gradually addressing these challenges to increase the widespread application of 4D bioprinting in regenerative medicine and tissue engineering. As discussed in the following section, incorporating novel materials such as self-assembling nanomaterials within bioprinting can be used to address some of the current challenges with 4D bioprinting such as poor cell adhesion and viability.

## 6. Future perspectives

As outlined above, many of the current materials used for 4D bioprinting applications lack desired characteristics such as quality printability, biocompatibility, and mechanical properties that mimic those of native tissue. Additionally, many smart materials that are currently used only respond to one type of stimuli, which does not accurately replicate the multi-responsive nature of natural tissues or organs. To address these challenges, the use of biomaterials that respond to multiple types of stimuli must be optimized. Additionally, integrating cells derived from patients directly into bioprinting can be used to develop patient-specific models. Using a patient’s own cells can also prevent immune system rejection if the bioprinted tissue constructs were to be implanted *in vivo*. Finally, the optimization of multi-material bioprinters that can precisely print biomaterials with different viscosities and smart behaviors is necessary to advance the field of bioprinting. Materially speaking, future work lies in introducing novel materials into bioprinting to adequately address the challenges discussed. For example, self-assembling nanomaterials are an up-and-coming type of nanomaterial that have the potential to enhance the cytocompatibility and functionality of bioprinted constructs.

When fabricating biological constructs for tissue engineering applications, it is crucial to maintain proper cell adhesion and proliferation within the printed scaffolds. Incorporation of self-assembling nanomaterials into bioprinted constructs has recently been studied as a means to promote cell adhesion, ultimately enabling tissue growth. Molecular self-assembly refers to the spontaneous binding of molecules under thermodynamic conditions into a stable supramolecule via non-covalent interactions [112]. These interactions maintain the stability of molecules at a low-energy state [112]. The unique properties of self-assembly nanomaterials offer exciting opportunities in the fields of nanomedicine. For example, self-assembling nanomaterials with injectable properties are being studied as a novel way to deliver therapeutic agents and promote tissue regeneration in hard-to-reach anatomical locations [113–116]. Incorporating these materials into bioprinting, which has enhanced reproducibility and cell spatial patterning compared to traditional scaffold fabrication methods, has the potential to help fabricate constructs that more accurately mimic the functionality of native tissue. In this section, both peptide and DNA-based self-assembly nanomaterials are discussed in addition to their potential applications in the field of bioprinting. Although only self-assembling nanomaterials are discussed, it is crucial to note that combinations of novel biomaterials such as these are necessary in order to advance and support 4D bioprinting processes.

**Figure 9.**
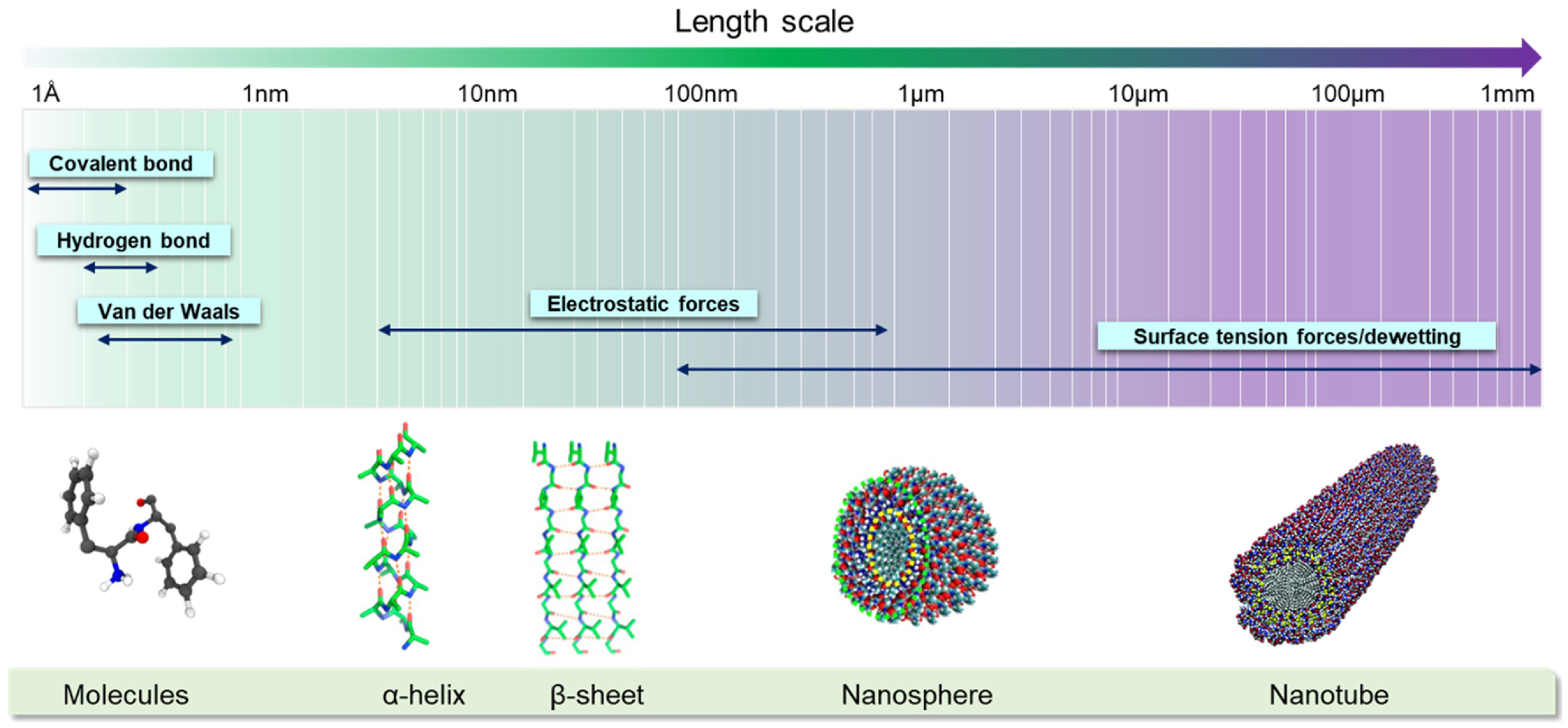
The types of interactions involved in peptide-based self-assembly and the resulting nanostructures. Reproduced with permission from [112]. CC BY-NC-ND 4.0.

Peptide-based nanomaterials spontaneously assemble from peptide monomers, forming ordered and thermodynamically stable supramolecules [117]. The spontaneous assembly of individual peptide monomers into organized structures is mainly controlled by non-covalent interactions, such as hydrogen bonding, van der Waals forces, and electrostatic interactions, as seen in figure 9 [112]. Although individual non-covalent bonds are weak compared to covalent bonds, the synergistic effect of non-covalent interactions allows for the formation of thermodynamically stable nanostructures with minimum energy states [117]. In a ‘bottom-up’ self-assembly process, peptide molecules containing hydrophobic and hydrophilic amino acid sequences interact with each other to form nanostructures of varying dimensions, including nanofibers, nanotubes, and nanospheres [117]. In addition to intrinsic factors such as the sequence of the peptides, the number of the amino acids, and the properties of the side chains, various external factors such as pH and temperature can affect the morphology of peptide-based self-assembled structures as well, making them promising smart biomaterials [112]. Unlike synthetic polymers which are denoted by non-uniform chain lengths and weight distributions, proteins consist of uniform peptide lengths, allowing for the formation of structurally ordered biomaterials [118]. Ordered peptide-based self-assembly nanomaterials have many advantages such as excellent biocompatibility, semiconductivity, and piezoelectricity [112]. Additionally, peptides can be modified with stimuli-responsive triggers that respond to various conditions, allowing for control over the assembly and disassembly of the overall structure. Different stimuli have been used to regulate the peptide assembly process including pH, where its fluctuation can change the charge of functional groups on peptide chains in aqueous solution, resulting in an attractive-repulsive transition of electrostatic forces [117]. On the other hand, high temperatures reduce the number of hydrogen bonds, consequently decreasing the strength of the interaction and the stability of the structure [117].

The biocompatible and versatile nature of peptide-based self-assembly nanomaterials has made them promising novel biomaterials within the field of tissue engineering. Due to their biocompatible nature, scaffolds constructed by self-assembling peptides are ideal environments for cell adhesion, migration, and proliferation [118]. Peptide-based self-assembly nanomaterials are commonly employed for hard-tissue engineering applications due to their ability to imitate the biomineralization of bone [119]. For example, Eren *et al* used negatively charged amphiphilic self-assembling peptide amphiphile molecules as a scaffold to induce calcium phosphate mineralization and enhanced osteogenic differentiation [120]. The mineral deposition ultimately enhanced the bioactivity of the peptide-based scaffold environments, promoting bone regeneration [120]. Similarly, Chen and Webster chemically functionalized short peptides of bone morphogenic protein-7 onto nanoscale biomaterials to enhance osteoblast proliferation and calcium deposition, ultimately promoting bone regeneration [121]. Due to their injectable nature, the stable scaffolds formed by peptide-based self-assembly nanomaterials can also be used to promote tissue regeneration in hard-to-reach anatomical locations, as seen in Smith *et al* where an injectable, multiphase transitioning peptide hydrogel was developed for suturing micro-scale vessels [122]. Future work in the field of 4D bioprinting involves incorporating peptide-based self-assembly nanomaterials, such as the ones mentioned, into 4D-bioprinted scaffolds, ultimately promoting stimuli-responsive tissue regeneration with enhanced cell adhesion and proliferation. However, there are some setbacks in utilizing protein-based nanomaterials. For example, the inherently unstable and complex nature of proteins has introduced some limitations when using peptide-based self-assembly nanoparticles for clinical applications [123]. Therefore, modifications must be made to enhance the stability of peptide-based self-assembly nanomaterials prior to widespread clinical use.

DNA has many advantageous properties including its minute size and information-storing capabilities. DNA-based assemblies also have well-known structural properties, which allows users to organize nanoparticles within the self-assembled scaffolds with great specificity to construct supramolecular materials [124]. In addition to their programmability and stability, DNA-inspired self-assembly nanomaterials can also be integrated into stimuli-responsive systems. Specific DNA sequences possess stimuli-responsive properties, which can promote further control of DNA nanostructures. For example, guanine sequences can fold into G-quadruplex structures in the presence of metal ions such as potassium and sodium [125]. Additionally, acidic pH triggers the assembly of cytosine-rich sequences into i-motif structures, which are highly dynamic DNA secondary structures that are thought to help with gene regulation and expression [125].

**Figure 10.**
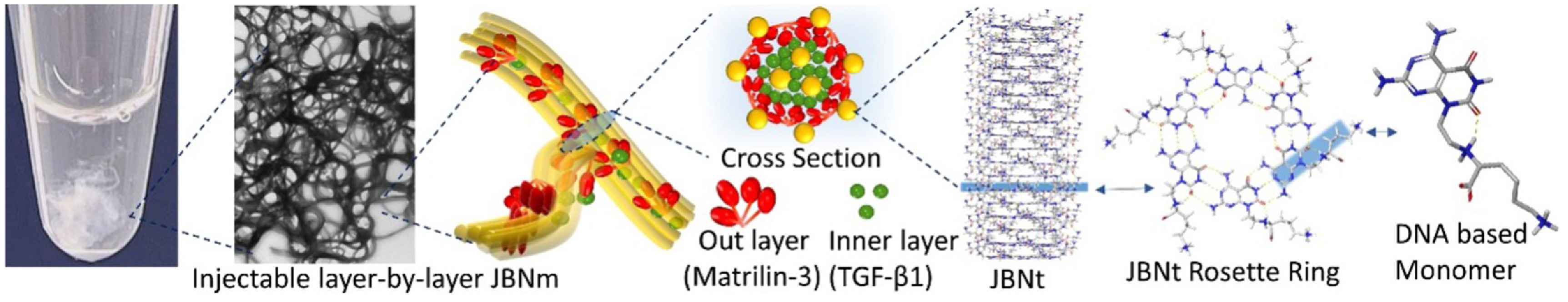
Self-assembly of DNA-inspired nanomaterials termed JBNms. Reproduced with permission from [129]. CC BY-NC-ND 4.0.

The customizable and stimuli-responsive nature of DNA self-assembly nanomaterials has made them promising additives for many biological applications. Due to its biocompatibility, the scaffolds formed from DNA-inspired self-assembly nanomaterials can be utilized to promote cell adhesion and proliferation, ultimately promoting tissue regeneration. For example, Zhou *et al* developed a biomimetic ECM-like scaffold termed a Janus base nano-matrix (JBNm), which has been determined to enhance stem cell anchorage [126, 127]. The scaffold is formed through the assembly of fibronectin, an adhesive glycoprotein, and DNA-inspired Janus base nanotubes (JBNts) [126]. JBNts, which form the JBNm backbone, self-assemble from small molecule building blocks derived from GC or AT DNA base pairs, forming a stable structure via hydrogen bonds, *π*-stacking interactions, and hydrophobic effects [128, 129]. Since JBNts and many ECM molecules are oppositely charged, when combined, they bond through non-covalent interactions and self-assemble to form JBNm bundles, as seen in figure 10 [126, 129].

Nanomaterials like JBNts and JBNms are favorable for biological applications since they can self-assemble *in situ* without the presence of harsh chemical initiators [128]. Additionally, they have been shown to promote enhanced cell adhesion and proliferation while also encouraging stem cell differentiation [128]. Furthermore, JBNts were shown to promote RNA delivery and tissue regeneration in hard-to-reach anatomical locations [130–132]. For example, Chen *et al* combined rosette nanotubes (a type of JBNts), which self-assembled from DNA base pairs, with hydrogels and cells via electrospinning to generate 3D implantable scaffolds for cartilage regeneration [115]. Not only did the JBNt/hydrogel composites show favorable cell chondrogenic differentiation, but since JBNts mimic the natural structure of collagen, they also demonstrated enhanced hydrogel adhesive strength to damaged collagen, promoting targeted cartilage repair [115, 133]. By modifying JBNms with different proteins and growth factors, the scaffolds can be tailored to various types of cell differentiation [134]. This nanotechnology has the potential to overcome the current limitation of 3D bioprinting complex biological materials in a timely manner. For example, a JBNm (formed from matrilin proteins and JBNts) can be fabricated into a layer-by-layer structure via controlled self-assembly at the molecular level in seconds through hydrogen bonds (figure 10) [129]. Since JBNts are electroconductive due to their molecular structure, JBNms also have the potential to be integrated into electro-responsive 4D bioprinting applications [135]. Ultimately, due to their versatile and biocompatible nature, the integration of DNA-inspired self-assembly nanomaterials into bioprinted constructs is being investigated as a novel way to enhance tissue regeneration *in vivo,* especially with pH or temperature stimuli.

## 7. Conclusion

Overall, significant advancements in the field of bioprinting have been achieved over recent years. Although 3D bioprinting has been used to successfully fabricate biological scaffolds for *in vitro* modeling purposes, the constructs fail to accurately mimic the dynamic nature of native tissue. The 4D bioprinting has recently emerged as a novel way to fabricate biomaterials with stimuli-responsive properties, allowing researchers to gain a better understanding of the functionality of tissue *in vivo*. Various types of stimuli-responsive biomaterials, including physical-, chemical-, and biological-sensitive materials were discussed in this review in addition to their wide range of tissue regeneration applications. However, several challenges have halted the widespread use of 4D bioprinting for clinical applications. For example, many stimuli-responsive biomaterials can only perform simple shape changes and they oftentimes lose their unique structural properties after repeated deformations. Additionally, some stimuli-responsive materials require the presence of harsh chemicals to initiate structural transformations, which can be harmful to surrounding tissues if used in clinical applications. Stimuli-responsive biomaterials that lack bioactivity may also prevent proper cell adhesion and proliferation, reducing overall cell viability. The integration of self-assembling nanomaterials within bioprinted constructs could ultimately address these challenges, revolutionizing the field of 4D bioprinting. Due to their controllable and versatile nature, these types of nanomaterials have the potential to promote more complex shape transformations of bioprinted constructs. Additionally, both peptide-based and DNA-based self-assembling nanomaterials have been successfully used as scaffolds to promote cell adhesion, therefore, incorporation of these materials within bioprinting could enhance cell growth. The injectable and biocompatible nature of these nanomaterials would also allow them to be easily incorporated into bioink prior to bioprinting. Future work in this field lies in enhancing the stability of the nanomaterials and studying their compatibility with various smart biomaterials currently used in bioprinting. Ultimately, 4D bioprinting is an advancing field that’s emerging as a promising approach to fabricate functional constructs for tissue regeneration applications.

## Data Availability

All data that support the findings of this study are included within the article (and any supplementary files).
